# Determinants of agility in youth basketball: age-stratified hierarchical regression in 6–13-year-old boys

**DOI:** 10.1186/s13102-026-01594-z

**Published:** 2026-03-09

**Authors:** Gizem Başkaya, Veli Volkan Gürses, Serkan Necati Metin, Ömer Özer, Kamil Uzgur, Ozan Burak Akduman, Sare Dündar, Ali Erdem Ciğerci, Yağmur Akkoyunlu, Ali Özkan

**Affiliations:** 1 Bandırma Onyedi Eylül University, Faculty of Sport Sciences, Balıkesir, Turkey; 2 Bandırma Onyedi Eylül University, Manyas Vocational School, Balıkesir, Turkey; 3 Kastamonu University, Faculty of Sport Sciences, Kastamonu, Turkey; 4 Yozgat Bozok University, Faculty of Sport Sciences, Yozgat, Turkey

**Keywords:** Youth basketball, Agility, Change-of-direction, Sprint speed, Vertical jump, Hexagon test, Triponderal mass index

## Abstract

**Background:**

Agility in youth basketball reflects the interplay between body dimensions and motor abilities. Age-specific prediction models guide age appropriate training design and evidence based talent identification.

**Methods:**

Ninety-eight male players (6–13 y) from a basketball school were classified into 6–9 y (*n* = 44) and 10–13 y (*n* = 54) groups. Anthropometry included height, mass, and the triponderal mass index (TMI). Motor performance comprised the 20 m sprint, countermovement jump (CMJ), and the Hexagon test. Agility was assessed with the V-CUT test. Pearson (and, where normality was violated, Spearman) correlations were computed; age-stratified hierarchical regressions identified determinants of V-CUT. Hierarchical regression was performed by adding TMI, 20 m sprint, CMJ, and Hexagon, respectively.

**Results:**

V-CUT time correlated strongly and positively with 20 m sprint in both groups (6–9 y: r = .807, *p* < .001; 10–13 y: r = .619, *p* < .001) and moderately and negatively with CMJ (6–9 y: r = − .440, *p* = .001; 10–13 y: r = − .337, *p* = .007). Associations with TMI were small and non-significant. In regression, adding 20 m sprint markedly increased explained variance (6–9 y: R^2^ = .657; 10–13 y: R^2^ = .387, both *p* < .001). Final models yielded R^2^ = .659 (6–9 y) and R^2^ = .476 (10–13 y); Hexagon provided additional unique variance only in the older group (ΔR^2^ = .082, *p* = .009), whereas CMJ contributed minimally once sprint was entered.

**Conclusion:**

Sprint speed is the primary determinant of agility (V-CUT) in young basketball players, while multidirectional change-of-direction ability (Hexagon) gains importance from 10–13 y. Anthropometric indices show limited predictive value. These results support emphasizing early sprint development and progressively integrating multidirectional drills in older athletes to inform age-appropriate training and talent identification.

## Introduction

Basketball is one of the most widely played team sports worldwide, engaging millions of young athletes in recreational and competitive contexts. Its dynamic nature requires frequent accelerations, decelerations, and rapid direction changes, making agility a fundamental determinant of performance. These demands make agility a critical determinant of performance, particularly in youth athletes during early developmental stages when physical growth and motor learning interact dynamically [1, 2]. In basketball, agility is expressed through sport-specific technical actions such as defensive slides and close-outs, rapid cuts to create space or receive passes, change-of-pace dribbling, and transition accelerations during offense–defense turnovers. These actions require efficient braking and re-acceleration, trunk stability, and coordinated footwork under time pressure. The relative contribution of these components may differ between early childhood (6–9 years), when fundamental movement skills and coordination dominate, and preadolescence (10–13 years), when increases in strength, sprint capacity, and tactical complexity place greater emphasis on faster and more precise change-of-direction execution [3–6].

The ability to rapidly change direction or velocity in response to a stimulus while maintaining balance and control [3, 7–9]. Contemporary models conceptualize agility as a multifactorial construct influenced by neuromuscular coordination, biomechanical efficiency, and cognitive-perceptual processes [8, 10–12]. Within this framework, both anthropometric characteristics (e.g., body height, mass, limb length, and segmental ratios) and motor performance attributes (e.g., sprint speed, vertical jump performance; CMJ height, and multidirectional movement capacity) are recognized as key contributors to agility outcomes in youth athletes [13–16]. Understanding the determinants of agility in youth basketball has become a central focus for sports scientists, coaches, and talent development programs [3–6]. In recent years, sports scientists and coaches have increasingly emphasized the importance of objective and field-based assessments to predict agility and inform individualized training programs for young athletes [1, 17].

Over the last decade, numerous studies have examined how anthropometric and motor variables relate to agility and change-of-direction performance in youth sports. Among motor predictors, recent studies have reported moderate to strong associations between sprint performance and agility across sports, including basketball, soccer, and tennis [18, 19]. CMJ height and reactive strength have also been linked to agility through their reliance on stretch–shortening cycle efficiency [6, 20, 21]. In contrast, anthropometric measures such as body mass index (BMI) and triponderal mass index (TMI) show mixed associations, with some evidence suggesting that excessive body mass may hinder change-of-direction performance [22]. In addition to BMI, TMI (weight/height^3^) has recently been proposed as a more stable indicator of adiposity in pediatric populations [23–25]. Given these advantages, TMI was selected in the present study as a complementary morphological parameter to BMI in predicting agility performance.

Despite these insights, significant gaps remain. Most studies focus on single predictors rather than integrating multiple anthropometric and motor variables into regression-based models, limiting their practical value for training and talent identification [26]. Furthermore, despite significant differences in biological maturation and neuromuscular development between early childhood and early adolescence, there are very few studies examining age-specific determinants of agility [1, 4, 9, 27, 28], and there is still a need for research in the literature that aims to highlight these differences. Finally, validated field tests such as the V-CUT and Hexagon protocols remain underutilized in predictive modeling, despite their reliability and ecological relevance [18, 29]. In this manuscript, the term ‘agility’ is used strictly to denote preplanned change-of-direction speed (CODS), operationalized by V-CUT performance. In contrast, the Hexagon test is treated as an indicator of multidirectional coordination and movement skill rather than an agility measure, and it is therefore discussed separately from preplanned CODS outcomes [7].

Methodological limitations also persist in literature. Many studies use small, homogeneous samples, limit generalizability, and overlook confounding factors such as training experience, maturation, or positional roles [9, 15, 26, 28, 30]. While laboratory assessments provide precision, they lack ecological validity compared to field-based contexts where agility occurs in real competition. Addressing these shortcomings is essential for developing practical, evidence-based tools to guide individualized training and long-term athlete development. Given this need, research should adopt regression-based models to determine the relative contribution of multiple predictors while accounting for age-specific developmental differences. Such approaches would advance theoretical understanding and provide actionable insights for coaches and talent identification systems.

Therefore, grounded in the Sheppard-Young model of agility (neuromuscular and perceptual components), we examined age-specific predictors of preplanned change-of-direction performance (V-CUT) in male basketball players aged 6–13 years. Additionally, by addressing developmental differences in the determinants of agility, we aimed to determine whether determinants differed between two age groups (6–9 and 10–13 years old). We hypothesized that: (H1) linear sprint would be the dominant determinants across ages; (H2) multidirectional coordination (Hexagon) would contribute more in the older group; and (H3) anthropometric indices (including TMI) would show limited predictive value after motor variables. This study extends previous research by combining age-specific regression modeling with the use of the triponderal mass index (TMI) to examine developmental differences in agility-related performance among youth basketball players.

## Methods

### Study design

This study used a cross-sectional design to examine the determinants of agility performance using anthropometric and motor variables, aiming to develop regression models for youth basketball players. The sample was stratified into two age groups (6–9 years and 10–13 years) to account for developmental differences. Athletes in both age groups train for a total of 180 min per week, with two 90-min sessions. Selected participants, coaches and parents were provided with detailed information about the purpose, duration and requirements of the training, and the necessary approvals were obtained from the participants' families for them to take part. All assessments were scheduled on two consecutive test days at the same time of day (10:00–12:00) on an indoor hardwood court (23 °C ± 1 °C) with athletes wearing court shoes. To control fatigue related confounding, tests were sequenced a priori from lower to higher neuromuscular demand: Anthropometry, CMJ, and the 20 m sprint were conducted on Day 1, while the Hexagon and V-CUT tests were completed on Day 2. Two trials were performed for each test with a 2-min rest interval, and the best value was retained for analysis. All assessments were administered by the same trained investigator using standardized procedures. Participants were instructed to maintain their usual diet, refrain from strenuous physical activity on testing days, and attend testing at least two hours after meals. All baseline assessments were conducted two hours after meals, and participants were instructed to refrain from physical exercise on the day of testing. Before data collection, all participants were carefully screened to support and emphasize eligibility based on predefined inclusion and exclusion criteria. Although biological maturation indicators such as peak height velocity (PHV) are recognized as important determinants of neuromuscular performance in youth, they were not assessed in the present study due to the field-based nature of data collection and the absence of longitudinal anthropometric data required for maturity offset estimation. To partially account for developmental differences, participants were stratified into two age groups and all analyses were conducted separately by age. This methodological approach was designed to generate a practical and field-based predictive framework for understanding the relationship between anthropometric and motor performance variables and agility in youth basketball players.

### Participants

A priori power analysis was conducted using G*Power 3.1.9.2. Assuming three main determinants, the analysis indicated that a minimum of 77 participants would be required to achieve a statistical power of 0.80 at α = 0.05, effect size *f*^*2*^ = 0.15. Therefore, the sample size of 98 athletes in this study exceeded the required threshold.

A total of 98 healthy male children participated in the study. All participants voluntarily took part, and because they were underage, written informed consent was obtained from their parents or legal guardians. Inclusion criteria were defined as healthy male basketball players aged between 6–9 (*n* = 44) and 10–13 (*n* = 54) years. Exclusion criteria included any known chronic diseases, musculoskeletal disorders, or cardiovascular conditions. To determine eligibility, participants were asked to complete a standardized health screening questionnaire, including family medical history. In addition, the researchers confirmed both players' and parents' statements through individual interviews [31, 32]. No participant presenting symptoms related to the exclusion criteria was included in the study.

### Data collection tools

#### Anthropometric measurements

Anthropometric variables were assessed following standardized guidelines to ensure precision, validity, and reliability. Body height was measured using a stadiometer (Seca 213; Seca GmbH & Co. KG, Hamburg, Germany) with 0.1 cm accuracy, and body mass was recorded with a segmental body composition monitor (TANITA BC-558 Ironman, Tanita Corporation, Tokyo, Japan) with 0.05 kg accuracy. Participants were measured barefoot and wearing only light clothing. BMI was calculated as body mass (kg)/height (m^2^), and TMI was computed as body mass (kg)/height^3^ (m^3^). Arm and hand span were measured using a flexible anthropometric tape (Seca 201, Seca GmbH & Co. KG, Hamburg, Germany). All anthropometric assessments were performed twice, and the best measure was retained for analysis.

#### V-CUT Agility Test (V-CUT)

Agility performance was measured using the V-CUT test, a widely applied protocol in basketball to assess change-of-direction ability [1, 17]. The test was conducted on a standard basketball court (28 m × 15 m, parquet flooring) located in an indoor sports hall. The test was performed over a 25 m course with four directional changes of 45° every 5 m. Players started between two cones set 0.7 m apart, sprinted through the course, and finished between two cones at the end. Each participant performed two attempts, separated by two minutes of passive rest. Times were recorded to the nearest 0.01 s using electronic timing gates, and the best performance was used in the analyses [29]. The V-CUT test followed the protocol outlined by Gonzalo-Skok et al. [17], involving four 45° cuts. Due to court geometry constraints, a 25 m variant was used. To ensure comparability, a sensitivity analysis was conducted using z-standardized V-CUT times.

#### Hexagon test

The Hexagon test evaluated multidirectional agility and lower-limb coordination [33]. A hexagon (24 in per side, 120° angles) was marked on the floor with tape. Starting at the center, participants performed continuous two-footed jumps over each side for three complete circuits (18 jumps total), always returning to the center after each jump. Timing began with the starting signal and stopped when the athlete returned to the center at the end of the final circuit. The fastest of the two trials was recorded.

#### Countermovement Jump (CMJ)

Vertical jump performance was assessed using the CMJ test [14]. Measurements were taken using the MyJump 2 mobile application, whose validity and reliability have been established by various researchers (ICC = 0.997) [34–38]. For the CMJ, athletes were instructed to perform a rapid downward movement from the starting position (approximately 90° knee flexion), followed by a rapid upward movement to jump as high as possible. Participants performed two maximal jumps with arm swing permitted, and jump height (cm) was recorded. The best trial was retained for analysis. CMJ height has been widely used to indicate explosive lower-limb power in youth athletes [14].

#### 20 m sprint test

Sprint performance was assessed over 20 m using photocell timing gates (Witty System, Microgate, Bolzano, Italy) positioned 1 m above the ground at the start and finish lines. The players started standing 50 cm behind the first gate with their front feet close to the line. Twenty-meter sprint times were recorded using dual photocell gates positioned at a height of 1.0 m. A standardized standing-start position was adopted, with the lead forefoot placed directly behind the start line and hands free beside the trunk. Two attempts were performed, separated by 2 min of rest, and the fastest sprint time was recorded [18]. Lloyd et al. (2014) particularly emphasize the developmental change and suitability of tests to the natural movement repertoire in neuromuscular assessments of children and adolescents. Gallahue & Ozmun (2006) stress that children use all segments of movement together when exhibiting basic motor skills, and that upper extremity restriction disrupts the naturalness of the motor task. Therefore, arm-swing preserves the child's accustomed jumping pattern and cognitively simplifies the task demand [39, 40].

#### Health screening questionnaire

A specific pediatric Preparticipation Physical Examination (PPE) health history screening questionnaire and a brief semi-structured interview checklist modified from AHA pediatric screening guidance were developed to verify medical history, participation eligibility, and training background in children aged 6–13 years [31, 32]. Item content drew on established PPE recommendations and AHA pediatric screening guidance [41, 42]. Both tools were piloted to ensure clarity.

#### Determinant variables

Independent variables included anthropometric measures: Height, body mass, BMI, TMI, and motor performance outcomes (20 m sprint time, CMJ height, Hexagon test time). The dependent variable was agility performance, operationalized through the V-CUT test, expressed in seconds. Correlations were computed for all measures; regression models were a-priori restricted to TMI, 20 m sprint, CMJ, and Hexagon. Predictor variables were selected a priori to represent key and non-overlapping domains relevant to agility-related performance in youth. TMI was included as a concise indicator of body composition, while the 20 m sprint, CMJ, and Hexagon tests were chosen to capture linear speed, lower-limb power, and multidirectional coordination, respectively. Additional anthropometric variables were not included to minimize multicollinearity and maintain model parsimony.

### Statistical analysis

All data were analyzed using SPSS version 27.0 (IBM Corp., Armonk, NY, USA). Descriptive statistics were reported as means and standard deviations. Statistical significance was set at two-tailed α = 0.05. Distributions were screened with Shapiro–Wilk tests and Q-Q plots; when normality was violated, we reported Spearman’s ρ in addition to Pearson’s r (with 95% CIs from Fisher’s z) to describe bivariate associations with V-CUT.

The order of entry in the hierarchical regression models and all study hypotheses were defined a priori based on theoretical and empirical considerations. Hierarchical linear regression analyses were run within each age group using four a priori blocks to quantify incremental contributions; Block 1 Anthropometry (TMI), Block 2–20 m sprint, Block 3-CMJ, Block 4-Hexagon. Assumptions were checked on standardized residuals: linearity, normality, homoscedasticity and independence. Multicollinearity was inspected using VIF (acceptable if < 5). Anthropometry was represented by TMI to avoid redundancy with height and mass. We report unstandardized coefficients (B, standard error [SE], 95% CI, t, exact p), standardized coefficients (β), and model quality (R^2^, Adjusted R^2^, ΔR^2^ with Sig. F-change, standard error of the estimate (SEE), overall F with degrees of freedom, and Durbin-Watson. Analyses used complete-case data; influential observations were screened and did not alter inferences. Where computed, internal validation and model selection indices were additionally reported AIC/AICc from residual sum of squares (SSE), sample size (n), and parameter count (k). Training exposure was uniform (2 × 90 min/week); therefore, it was not entered into the models. Assumptions were verified on studentized residuals; influential points were inspected.

## Results

### Descriptive characteristics

Participants were 98 male basketball players aged 6–13 y (*n* = 44 in 6–9 y; *n* = 54 in 10–13 y). Descriptive statistics for anthropometry; body height, body mass, TMI, and performance V-CUT, 20 m sprint, CMJ and Hexagon by age group are presented in Tables 1 and 2. These values provide the context for the correlation and regression analyses that follow.

**Table 1:** Characteristics and performance variables of participants aged 6–9 years (*n* = 44)

Variables	Mean ± S.D	Min–Max	%95 Lower	%95 Upper
Age (years)	7.30 ± 1.12	6–9	6,99	7.61
Height (cm)	139.72 ± 10.87	114–165	136.79	142.74
Body mass (kg)	36.61 ± 11.14	17–63	33.29	40.02
TMI (kg/m^−3^)	13.16 ± 2.51	9.91–19.05	12.43	14.05
VCUT (s)	10.00 ± 1.07	8.07–12.61	9.68	10.30
Hexagon (s)	32.47 ± 7.10	23.62–50.00	30.57	34.66
CMJ (cm)	20.25 ± 6.57	9.47–35.49	18.56	22.11
20 m Sprint (s)	5.34 ± 0.56	4.32–6.81	5.17	5.51

Values are Mean ± SD with range (Min–Max)

For time variables (VCUT, Hexagon, 20-m sprint), lower values indicate better performance; for CMJ, higher values indicate better performance. Where reported, 95% CI refers to the confidence interval for the mean

*TMI* Triponderal mass index (kg·m⁻^3^), *VCUT* V-cut change-of-direction test time (s), *Hexagon* Hexagon agility test time (s), *CMJ* Countermovement jump height (cm), *20-m sprint* electronically timed 20-m sprint time (s)

**Table 2:** Characteristics and performance variables of participants aged 10–13 years (*n* = 54)

Variables	Mean ± S.D	Min–Max	%95 Lower	%95 Upper
Age (years)	11.52 ± 1.05	10–13	11.21	11.83
Height (cm)	145.21 ± 17.81	118–189	140.65	150.18
Body mass (kg)	41.37 ± 16.96	21–96	37.27	45.91
TMI (kg/m^−3^)	13.04 ± 2.21	8.54–18.31	12.45	13.56
VCUT (s)	9.24 ± 0.97	7.34–11.76	8.97	9.52
Hexagon (s)	18.42 ± 2.78	12.96–24.70	17.71	19.12
CMJ (cm)	20.51 ± 6.09	7.48–36.51	18.80	22.13
20 m Sprint (s)	5.19 ± 0.57	3.99–6.61	5.03	5.34

Values are Mean ± SD with range (Min–Max)

For time variables (VCUT, Hexagon, 20-m sprint), lower values indicate better performance; for CMJ, higher values indicate better performance. Where reported, 95% CI refers to the confidence interval for the mean

*TMI* Triponderal mass index (kg·m⁻^3^), *VCUT* V-cut change-of-direction test time (s), *Hexagon* Hexagon agility test time (s), *CMJ* Countermovement jump height (cm), *20-m sprint* electronically timed 20-m sprint time (s)

### Correlation analyses

Pearson's correlation coefficients revealed significant associations between motor performance tests and agility (Tables 3 and 4). In the 6–9 age group, V-CUT performance showed a robust positive correlation with 20 m sprint time (*r* = 0.807, *p* < 0.001) and a moderate negative correlation with CMJ height (*r* = −0.440, *p* = 0.001). A small but significant positive correlation was also observed with Hexagon performance (*r* = 0.318, *p* = 0.018). TMI showed a weak and non-significant association with agility (*r* = 0.130, *p* = 0.201). In the 10–13 age group, similar trends emerged. V-CUT performance correlated strongly with 20 m sprint time (*r* = 0.619, *p* < 0.001) and moderately with CMJ (*r* = −0.337, *p* = 0.007). However, correlations with TMI (*r* = 0.187, *p* = 0.092) and Hexagon performance (*r* = 0.156, *p* = 0.135) were small and non-significant.

**Table 3:** Pearson’s correlation coefficients between V-cut time and performance predictors in children aged 6–9 years old (*n* = 44)

**Predicted Variable Vcut**		**TMI** **(kg/m**^**3**^**)**	**20 m Sprint** **(sn)**	**CMJ** **(cm)**	**Hexagon** **(kg/m**^**2**^**)**
**VCUT**	r	.130	.807	-.440	.318
	*p*	.201	.000^**^	.001^**^	.018^*^

Values are Pearson’s r with two-tailed *p*-values showing associations between VCUT (s) and each predictor (TMI, 20 m sprint time, CMJ height, Hexagon time)

For time variables (VCUT, 20 m sprint, Hexagon), lower values indicate better performance; for CMJ, higher values indicate better performance. Statistical significance set at α = 0.05 (two-tailed)

*VCUT* V-cut agility test time (s), *TMI* Triponderal mass index (kg·m⁻^3^), *CMJ* Countermovement jump (cm), *Hexagon* Hexagon agility test time (s)

^*^p < 0.05

^**^ *p* < 0.01

^***^ *p* < 0.001

**Table 4:** Pearson’s correlation coefficients between V-cut time and performance predictors in children aged 10–13 years old (*n* = 54)

**Predicted Variable Vcut**		**TMI** **(kg/m**^**3**^**)**	**20 m Sprint** **(sn)**	**CMJ** **(cm)**	**Hexagon** **(kg/m**^**2**^**)**
VCUT	r	.187	.619	-.337	.156
	*p*	.092	.000***	.007*	.135

Values are Pearson’s r with two-tailed p-values showing associations between VCUT (s) and each predictor (TMI, 20 m sprint time, CMJ height, Hexagon time)

*Hexagon* Hexagon agility test time (s)

For time variables (VCUT, 20 m sprint, Hexagon), lower values indicate better performance; for CMJ, higher values indicate better performance. Statistical significance set at α = 0.05 (two-tailed)

*VCUT* V-cut agility test time (s), *TMI* Triponderal mass index (kg·m⁻^3^), *CMJ* Countermovement jump (cm),

^*^p < 0.05

^**^ *p* < 0.01

^***^ *p* < 0.001

### Hierarchical regression (6–9 y)

Entering TMI alone explained 1.7% of variance in V-CUT (R^2^ = 0.017, *p* = 0.402). Adding 20 m sprint increased explained variance to 65.7% (R^2^ = 0.657, *p* < 0.001). Subsequent inclusion of CMJ and Hexagon yielded negligible improvements (final model R^2^ = 0.659, Adj. R^2^ = 0.624, SEE = 0.658 s, DW = 2.181). In the final model, 20 m sprint remained the only substantial predictor; other coefficients were small and non-significant (Table 5).

**Table 5:** Hierarchical regression predicting VCUT (s) in 6–9 years old; Panel A-Coefficients; Panel B-Model fit (*n* = 44)

(A) Coefficients (final model—Block 4)										
Predictor	B	SE	95% CI (LL, UL)			t	p	β	Tolerance	VIF
Intercept	1.576	1.554	−1.567, 4.720			1.014	.317	-	-	-
20-m sprint (s)	1.596	0.230	1.131, 2.062			6.937	<.001	.834	.606	1.651
CMJ (cm)	0.006	0.019	−0.033, 0.045			0.315	.754	.037	.625	1.601
Hexagon (s)	0.006	0.015	−0.025, 0.036			0.378	.707	.038	.866	1.154
TMI (kg·m⁻^3^)	−0.031	0.042	−0.117, 0.054			−0.737	.465	-.073	.892	1.122

(B) Model fit (by blocks)										
Model/Block	**R**^**2**^	**Adj. R**^**2**^	**ΔR**^**2**^	**Sig. F-change**	**SEE (s)**	**F (df1, df2)**	**p**	**Durbin–Watson**	**AIC (full)**	**AICc (full)**
Block 1 (TMI)	.017	-.007	-	-	1.0769	0.717 (1,42)	.402	-	133.341	133.634
Block 2 (+ 20-m s)	.657	.640	.640	<.001	0.6440	39.234 (2,41)	<.001	-	89.029	89.629
Block 3 (+ CMJ)	.658	.632	.001	.777	0.6513	25.597 (3,40)	<.001	-	90.941	91.966
Block 4 (+ Hexago)	.659	.624	.001	.707	0.6584	18.822 (4,39)	<.001	2.181	92.780	94.359

Panel A reports unstandardized coefficients (B) with standard errors (SE), 95% confidence intervals (CI), t, exact p, and standardized coefficients (β). Collinearity is summarized by Tolerance and VIF. Panel B shows model fit by blocks: R^2^, Adjusted R^2^, ΔR^2^ (Sig. F-change), SEE (s), overall F with df, and Durbin–Watson. Time measures (VCUT, 20-m sprint, Hexagon) are in seconds (s); CMJ in centimeters (cm); TMI = triponderal mass index (kg·m⁻^3^). For values rounding to.000, report *p* <.001

Based on these findings, The final unstandardized equation was:

$$\begin{array}{ll}\mathrm V-\mathrm{CUT}\;\left(\mathrm s\right)\;&=1.576\;+1.596\;\left(20\;\mathrm m\;\mathrm{sprint},\;\mathrm s\right)\;\\&+0.006\cdot\mathrm{CMJ}\;\left(\mathrm{cm}\right)\\&+0.006\cdot\mathrm{Hexagon}\;\left(\mathrm s\right)\\&-0.031\cdot\mathrm{TMI}\;\left(\mathrm{kg}\cdot\mathrm m^{-3}\right).\end{array}$$

### Hierarchical regression (10–13 y)

TMI alone explained 3.5% of variance (R^2^ = 0.035, *p* = 0.183). Adding 20 m sprint increased R^2^ to 0.387 (*p* < 0.001). Including CMJ produced a minimal change (R^2^ = 0.394), whereas adding Hexagon further improved the model to R^2^ = 0.476 (Adj. R^2^ = 0.431, ΔR^2^ = 0.082, *p* = 0.009), indicating unique variance explained by Hexagon in older players. Final model fit indices were SEE = 0.734 s and DW = 2.264 *(*Table 6*)*.

**Table 6:** Hierarchical regression predicting VCUT (s) in 10–13 years old; Panel A-Coefficients; Panel B-Model fit (*n* = 54)

(A) Coefficients (final model—Block 4)										
Predictor	B	SE	95% CI (LL, UL)			t	p	β	Tolerance	VIF
Intercept	−0.199	1.993	−4.205, 3.807			−0.100	.921	-	-	-
20-m sprint (s)	1.352	0.250	0.849, 1.855			5.405	<.001	.791	.521	1.920
CMJ (cm)	0.028	0.023	−0.018, 0.074			1.212	.232	.173	.545	1.833
Hexagon (s)	0.104	0.038	0.028, 0.180			2.708	.009	.297	.929	1.076
TMI (kg·m⁻^3^)	−0.004	0.052	−0.109, 0.101			−0.077	.939	-.009	.816	1.226

(B) Model fit (by blocks)										
Model/Block	**R**^**2**^	**Adj. R**^**2**^	**ΔR**^**2**^	**Sig. F-change**	**SEE (s)**	**F (df1, df2)**	**p**	**Durbin–Watson**	**AIC (full)**	**AICc (full)**
Block 1 (TMI)	.035	.016	-	-	0.9662	1.819 (1, 50)	.183	-	145.952	146.197
Block 2 (+ 20-m s)	.387	.362	.352	<.001	0.7777	15.486 (2, 49)	<.001	-	124.338	124.838
Block 3 (+ CMJ)	.394	.356	.007	.466	0.7814	10.407 (3, 48)	<.001	-	125.755	126.607
Block 4 (+ Hexago)	.476	.431	.082	.009	0.7344	10.670 (4, 47)	<.001	2.264	120.214	121.518

Panel A reports unstandardized coefficients (B) with standard errors (SE), 95% confidence intervals (CI), t, exact p, and standardized coefficients (β). Collinearity is summarized by Tolerance and VIF. Panel B shows model fit by blocks: R^2^, Adjusted R^2^, ΔR^2^ (Sig. F-change), SEE (s), overall F with df, and Durbin–Watson. Time measures (VCUT, 20-m sprint, Hexagon) are in seconds (s); CMJ in centimeters (cm); TMI = triponderal mass index (kg·m⁻^3^). For values rounding to.000, report *p* <.001

The final unstandardized equation was:

$$\begin{array}{ll}\mathrm V-\mathrm{CUT}\;(\mathrm s)\;&=\;-0.199\;+\;1.352\cdot(20\;\mathrm m\;\mathrm{sprint},\;\mathrm s)\;\\&+\;0.028\cdot\mathrm{CMJ}\;(\mathrm{cm})\;+\;0.104\cdot\mathrm{Hexagon}\;(\mathrm s)\;\\&-\;0.004\cdot\mathrm{TMI}\;(\mathrm{kg}\cdot\mathrm m^{-3}).\end{array}$$

Sensitivity analyses using z-standardized V-CUT yielded the same inferential pattern as the primary models as direction and relative magnitude of coefficients and ΔR^2^ across steps, therefore supporting robustness; the summary results are provided in Tables 4 and 5. Across both age bands, 20 m sprint consistently explained the largest share of variance in V-CUT. CMJ related to V-CUT at the bivariate level but contributed little once sprint was in the model. Hexagon added explanatory power only in 10–13 y, suggesting an age-related rise in multidirectional coordination demands. TMI showed minimal predictive value in either group.

## Discussion

**Fig. 1:**
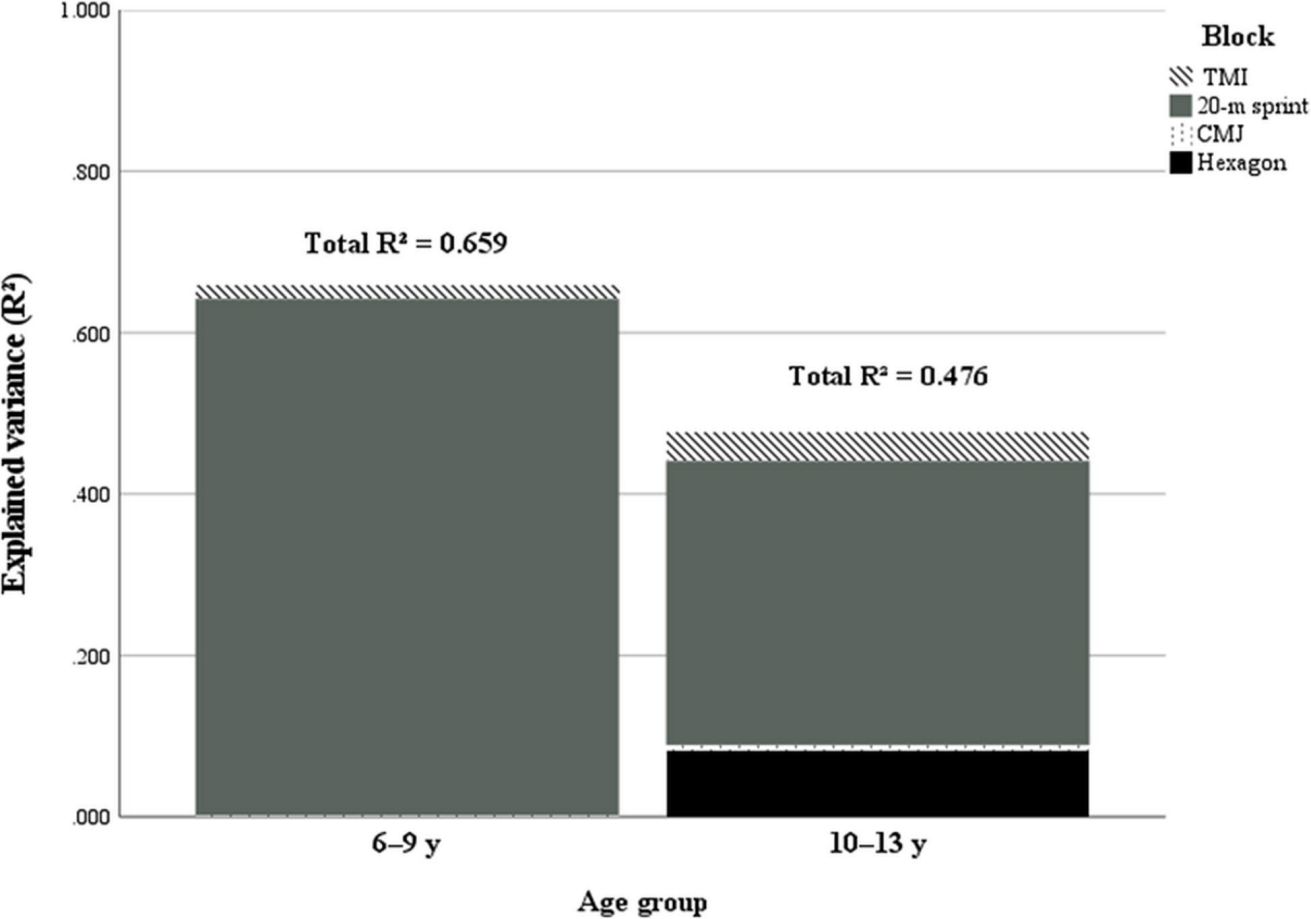
Explained variance by hierarchical blocks across age groups. Stacked bars show the incremental variance explained (ΔR^2^) by each block; TMI, 20-m sprint, CMJ, and Hexagon for the 6–9 y and 10–13 y models. Total model fit (Total R.^2^) is annotated above each bar. Sprint accounts for the largest share of explained variance in both groups; Hexagon adds unique variance only in 10–13 y, consistent with increasing multidirectional coordination demands. Values correspond to Tables 5 and 6

This study aimed to determine the anthropometric and motor determinants of agility in young basketball players by applying regression-based models in two age groups (6–9 years and 10–13 years). The results revealed that sprint speed was a reliable predictor of agility in both age groups, while anthropometric indices and vertical jump performance contributed very little. Furthermore, multidirectional coordination, measured using the Hexagon test, emerged as an additional determinants in older athletes (Fig. 1).

### Sprint as the primary determinant of agility

Sprint speed accounted for 65.7% of the variance in younger players (aged 6–9) and 38.7% of the variance in the older group (aged 10–13). Additionally, it has been demonstrated that sprint performance showed positive, moderate to high correlations with agility in both age groups (6–9 years: r = 0.807; 10–13 years: r = 0.619, *p* < 0.001). This finding aligns with a substantial body of literature reporting moderate to strong correlations between sprint ability and change-of-direction performance in team sports [18, 19, 30]. Negra et al. (2017) reported large to substantial correlations between sprint tests and agility outcomes (0.53 < r < 0.85, p < 0.001; shared variance 28–72%) [20]. Linear sprinting and agility share similar neuromuscular and biomechanical processes, such as force production, ground contact efficiency (short ground contact times), efficient lower limb mechanics, and lower limb power, which may explain the strong relationship between them [27]. Similar trends were reported by Horička & Šimonek (2019), who showed that acceleration (ACC-3 m) accounted for the largest share (71.5%) of reactive agility variance in basketball players [43]. The findings indicate that sprint development may be a priority component in the training process for young athletes during sensitive developmental stages [3, 9, 44].

CMJ and Hexagon performance emerged only as secondary, developmentally dependent predictors. Specifically, CMJ showed small but significant associations, likely reflecting overlapping contributions of explosive strength and stretch–shortening cycle efficiency, while the Hexagon test became a more relevant predictor in older players (10–13 age), indicating the growing role of multidirectional coordination with maturation [1, 21]. This streamlined interpretation emphasizes sprinting as the primary determinant of agility, with CMJ and Hexagon offering additional, context-dependent contributions. Collectively, these findings highlight sprinting as the primary determinant of V-CUT performance; however, sprint speed alone does not fully capture the rapid braking–re-acceleration demands of change-of-direction tasks. Accordingly, the next section considers the contribution of explosive power and neuromuscular coordination.

### Role of explosive power, vertical jump, and agility

Vertical jump performance showed moderate associations with agility, consistent with previous evidence highlighting the role of stretch–shortening cycle efficiency and explosive power in rapid directional changes [14, 20, 21]. However, in our regression models, its predictive contribution was marginal once sprint speed was included, indicating overlapping neuromuscular demands between sprinting and jumping [11, 15, 45–47]. This suggests that while sprint speed remains the dominant predictor, lower-limb explosive strength contributes meaningfully to agility, particularly in tasks requiring deceleration and reacceleration.

Age-specific analyses revealed further nuances. In the 6–9 age group, adding CMJ and Hexagon test performance to the regression model produced only marginal improvements in explained variance, while in the 10–13 group, Hexagon test performance contributed more substantially (increased the explained variance to 47.6%). Nevertheless, the Hexagon test is a closed-skill, preplanned movement sequence and does not capture the perceptual–cognitive demands of reactive agility; thus, its contribution should be interpreted as reflecting multidirectional coordination rather than stimulus-driven agility. This pattern suggests developmental differences in how multidirectional motor tasks interact with agility. Indeed, small but significant correlations were observed between V-CUT and Hexagon test performance in the younger cohort (6–9 age), whereas CMJ was negatively correlated with agility across both age groups (6–9 age: r = −0.440; 10–13 age: r = −0.337). These findings are consistent with prior studies reporting negative associations between jump test performance and agility times, reflecting that greater explosive power reduces time-to-completion in agility tasks [19, 20]. Given the rapid neuromotor reorganization around peak height velocity, the older group’s (10–13 age) unique Hexagon contribution likely reflects maturation-related gains in inter-segmental coordination and postural control during multidirectional tasks [11, 46, 48–50].

Biomechanically, this link is supported by the sequence of eccentric, isometric, and concentric contractions inherent to the stretch–shortening cycle, which mirrors the stop-start characteristics of agility movements [7, 51, 52]. While CMJ height clearly reflects explosive leg strength, its contribution to predicting agility outcomes appears secondary once sprint performance is accounted for. This aligns with training studies demonstrating that improvements in vertical or squat jump ability yield modest gains in agility, whereas targeted sprint and multidirectional speed training elicit more pronounced effects [53, 54]. Considered as a whole, these results suggest that although explosive power contributes meaningfully to agility, its role is conditional and developmentally dependent. Coaches should focus on sprint training as the primary determinant of agility in younger players, while progressively integrating plyometric and multidirectional drills to enhance explosive strength and movement efficiency in older cohorts. Future work should explore how maturation, sex differences, and perceptual-cognitive factors interact with neuromuscular performance to shape agility trajectories during adolescence [55]. While these results emphasize neuromuscular determinants, body size and mass distribution may still influence sprinting and change-of-direction mechanics. Therefore, we also examined whether anthropometric indices, particularly TMI, provide additional explanatory value beyond motor performance measures.

### Anthropometric indices and morphological parameters

In both cases, TMI alone contributed minimally, while the inclusion of sprint performance substantially increased the predictive value of the models. Contrary to expectations, anthropometric indices such as BMI and TMI accounted for only minimal variance in agility performance. This result supports findings by Muniroglu and Subak (2018), who reported weak associations between morphological traits and agility in children [56]. On the other hand, although previous studies have emphasised that excessive body mass may impair deceleration and re-acceleration [22], the relatively homogeneous and healthy sample in this study may have minimised the effect of anthropometric variation. In relatively homogeneous youth cohorts, anthropometric indices are therefore expected to add little explanatory power once motor variables are entered.

Developmental differences within the studied cohorts may explain the absence of strong associations between triponderal index and agility. Participants in our sample were in the pre-peak height velocity stage, where motor and physiological maturation are ongoing. Pavlinovic et al. (2022a) reported similar findings, attributing the weak associations partly to short-duration test protocols that may not adequately capture the metabolic demands of greater body or fat mass [19]. Other studies have likewise noted inconsistent or negligible relationships between children's anthropometric/body composition indices and preplanned agility tests [30, 41]. Spasic et al. (2013) also noted that in early adolescent girls, reactive strength, and sprint ability, rather than morphology, explained most agility variance (30–64%) [42]. These findings support the conclusion that functional motor skills are more important than morphological parameters in shaping agility performance during childhood and indicate that anthropometric/body composition indices cannot be reliable predictors of agility. To provide a context these age-specific findings, we next compare our results with prior basketball and youth-athlete studies that have examined similar field tests and predictor models.

### Agility determinants in basketball: evidence from comparative studies

Although our sample differed in age from some prior investigations, comparable studies in basketball provide valuable insights. Horička and Šimonek (2019) examined female players (mean age 21.7 years) and found that acceleration (ACC-3 m) accounted for the most significant proportion of variance (71.5%) in reactive agility, underscoring the centrality of sprint ability [43]. Their results also highlighted significant correlations between V-CUT and Hexagon-type agility tests, consistent with the associations observed in our study. They concluded that change-of-direction speed (CODS) and reactive agility should be considered distinct skills, with the relative contribution of motor determinants decreasing as task complexity increases, while cognitive demands become more influential.

Similarly, Pavlinovic et al. (2022a) investigated boys and girls aged 11–12 using the Triangle Reactive Agility Test (TRAG) [19]. While anthropometric and body composition indices showed negligible correlations with agility (0–4% shared variance), motor skills such as sprinting, broad jump, and CMJ were significant predictors (7–43% shared variance). In boys, CODS (Triangle-CODS) alone explained 64% of TRAG variance, highlighting again the primacy of motor over morphological factors. These discrepancies with our findings likely reflect methodological differences, as reactive agility protocols incorporate more complex perceptual and decision-making components than preplanned CODS tests used in our study.

Other studies reinforce this perspective. Spasic et al. (2013) reported that in early adolescent girls (12–13 years), reactive strength and sprint ability were stronger predictors of agility performance than anthropometric traits, with regression models explaining 30–64% of variance across multiple agility tests [42]. França et al. (2022) further showed that lower-body explosive power, assessed by squat jump, predicted agility outcomes more strongly than sprinting in some cohorts [57]. Interestingly, they observed that correlations between explosive power, speed, and agility declined with increasing chronological age, suggesting that maturation moderates these relationships.

Our findings partially align with these trends. In our cohort, sprint performance strongly correlated with agility in the younger group, but was only moderately associated with older players. This attenuation may reflect the influence of growth and biological maturation, as developmental status increasingly shapes agility outcomes during early adolescence. Indeed, prior research has emphasized that the ages 11–14 represent a sensitive period marked by rapid physical and biological change, intensified training exposure, and sport specialization [11, 46, 48–50]. These factors likely contribute to variability in agility predictors across age groups and highlight the importance of accounting for maturational status when interpreting youth performance data.

### Conclusion and recommendations

Overall, this study provides novel evidence that sprint performance is the primary determinants of agility in young basketball players. At the same time, age-related developmental differences modulate the contribution of additional motor variables. These findings offer valuable insights for talent identification and individualized training strategies in youth basketball, supporting evidence-based practices for coaches and practitioners.

Regression-based models incorporating TMI, 20 m sprint, vertical jump, and Hexagon performance successfully predicted agility in both age groups. In the 6–9-year-old players, TMI and 20 m sprint accounted for the most explained variance (65.7%), with vertical jump and Hexagon adding only marginal improvements. In contrast, in the 10–13-year-old group, the model including all four predictors explained 47.6% of variance, indicating a broader interplay of motor determinants at this developmental stage. These models may be supportive tools for predicting agility performance, though further refinement and validation are required before routine application. Consistent with our hypotheses, linear sprint emerged as the dominant determinants determinants in both age groups (H1), multidirectional coordination showed a comparatively greater contribution in the older group (H2), and anthropometric indices such as TMI added limited explanatory value once motor variables were considered (H3).

Importantly, relying solely on anthropometric and physical profiles to determine basketball ability may be insufficient as it overlooks cognitive-perceptual skills. As previous literature has emphasised the increasing role of cognitive abilities as agility tasks become more complex, future research should integrate perceptual-cognitive measurements and reactive agility assessments to improve prediction accuracy and inform more comprehensive training interventions.

### Practical implications

From a practical perspective, coaches working with players aged 6–9 should focus on short sprint accelerations (5–20 m), coordination ladder exercises, and tagging games that encourage quick starts and stops in a fun environment. These activities help develop neuromuscular coordination and fundamental movement skills, which are essential for future sport-specific performance.

Agility performance for players aged 10–13 is influenced by a broader set of variables, including multi-directional movement tasks such as the Hexagon test. Therefore, training for this age group should incorporate basketball-specific agility exercises. Examples include multi-directional short sprints, defensive slides combined with reactive cues (e.g., the coach's signal or the ball's movement), and 1v1 agility competitions where athletes must adapt to their opponent's movements. Furthermore, incorporating decision-making elements such as reacting to passing options or defensive rotations into agility training can better mimic the perceptual-cognitive demands of basketball match.

These recommendations indicate that sprint-based training should remain a fundamental component throughout all stages of development, but that basketball coaches should gradually integrate agility-focused and cognitively enriched exercises as athletes approach adolescence. This approach can enhance the transfer of training effects to on-court performance and support long-term athletic development. This progression may enhance the transfer of training effects to on-court performance and support long-term athletic development.

### Limitations and future directions

Despite its contributions, this study has several limitations. First, only healthy male basketball players were included. This limits the generalizability of the findings to female athletes and to children with health conditions. Accordingly, the external validity of the results is restricted to healthy male youth with comparable training exposure. In addition, sex- and age-specific physiological and anthropometric differences may influence test performance and, consequently, affect outcomes. Future studies should include biological maturation assessments (e.g., estimation of peak height velocity) to better account for growth-related variability in change-of-direction performance [58]. Including these factors would strengthen the ecological validity of predictive models and provide more comprehensive insights for coaches and talent identification programs. Study sample was restricted to players aged 6–13 years and divided into two categories: 6–9 years and 10–13 years. Training exposure and biological maturation were not precisely measured. These variables were not available in the dataset and thus could not be modeled as a separate hierarchical block. To partially mitigate confounding, analyses were stratified by age group and anthropometry was entered prior to motor variables to quantify incremental explanatory power. Future studies should implement PHV-adjusted mixed-effects models and incorporate reactive agility protocols to capture perceptual–cognitive demands that preplanned change-of-direction tests do not assess.We explicitly acknowledge the absence of training and maturity metrics as a limitation and a priority for future data collection. Future research should consider narrower age ranges, include more diverse populations, and incorporate assessments of maturity status to provide more precise information about developmental differences.

Secondly, as prior exposure to structured basketball training may influence motor skill efficiency and agility outcomes, the age at which training commences, and accumulated sporting experience should also be taken into account [59]. Another important avenue is the examination of basketball-specific positional roles (e.g., guard, forward, center), given that physical and agility demand vary across positions [28]. Including these factors would enhance the ecological validity of predictive models and provide more comprehensive insights for coaches and talent identification programs.

Thirdly, anthropometric measurements are subject to evaluator dependence and methodological limitations. While the tools used in this study were portable, inexpensive, non-invasive, and widely recognized as valid for estimating body composition, they may not capture regional variations as accurately as advanced techniques.

Fourth, agility was assessed exclusively using the V-CUT test and predicted by triponderal index, 20 m sprint, vertical jump, and Hexagon test performance. Although justified, this approach may have overlooked other important components of agility, particularly perceptual-cognitive and reactive elements. Current studies also encourage the integration of perceptual-cognitive measurements, such as reactive agility protocols, to capture the decision-making and foresight components that are of great importance in basketball [60, 61].

Finally, the study employed a cross-sectional design, which prevents causal inferences. Moreover, no internal validation techniques, such as k-fold cross-validation or bootstrapping, were applied, which may limit the generalizability of the regression models and risk overfitting. Future studies should adopt longitudinal designs and incorporate robust validation procedures to improve agility prediction models' reliability, stability, and practical applicability.

## Data Availability

The data sets used and/or analyzed during the current study are available from the corresponding author on reasonable request.

## Abbreviations

TMI: Triponderal Mass Index
CMJ: Countermovement Jump
V-CUT: V-CUT Test
BMI: Body Mass Index
cm: Centimeter
kg: Kilogram
m: Meter
VIF: Variance Inflation Factors
TRAG: Triangle Reactive Agility Test
CODS: Change of Direction Speed
SD: Standard Deviation
PHV: Peak Height Velocity
